# Spatial Difference-in-Differences with Bayesian Disease Mapping Models

**DOI:** 10.1097/EDE.0000000000001912

**Published:** 2025-09-10

**Authors:** Carl Bonander, Marta Blangiardo, Ulf Strömberg

**Affiliations:** From the a School of Public Health and Community Medicine, Institute of Medicine, University of Gothenburg, Gothenburg, Sweden; b Center for Societal Risk Research, Karlstad University, Karlstad, Sweden; c MRC Centre for Environment and Health, Department of Epidemiology & Biostatistics, School of Public Health, Imperial College London, London, United Kingdom.

**Keywords:** Causal inference, Spatiotemporal analysis, Spatial epidemiology, Quasi-experimental

## Abstract

Bayesian disease-mapping models are widely used in small-area epidemiology to account for spatial correlation and stabilize estimates through spatial smoothing. In contrast, difference-in-differences (DID) methods—commonly used to estimate treatment effects from observational panel data—typically ignore spatial dependence. This paper integrates disease-mapping models into an imputation-based DID framework to address spatially structured residual variation and improve precision in small-area evaluations. The approach builds on recent advances in causal panel data methods, including two-way Mundlak estimation, to enable causal identification equivalent to fixed effects DID while incorporating spatiotemporal random effects. We implement the method using Integrated Nested Laplace Approximation, which supports flexible spatial and temporal structures and efficient Bayesian computation. Simulations show that, when the spatiotemporal structure is correctly specified, the approach improves precision and interval coverage compared with standard DID methods. We illustrate the method by evaluating local ice cleat distribution programs in Swedish municipalities.

Credible estimates of intervention effects are essential for public health planning. Although randomized trials provide the strongest causal evidence, many interventions cannot be randomized due to practical or ethical constraints. Evaluating interventions as implemented in real-world settings is also valuable, as it captures the impact of contextual and implementation challenges. Consequently, many public health and epidemiologic evaluations rely on observational data, where causal inference is complicated by the risk of confounding bias.

Interest in spatial causal inference is growing in epidemiology,^1^ partly driven by the need to evaluate geographically targeted interventions, such as local road safety measures,^2^ hotspot policing,^3^ and healthcare initiatives in deprived areas.^4,5^ These interventions are often implemented based on local needs or political priorities, making randomization infeasible. Additionally, evaluating them typically requires high-resolution spatial data, which introduces further challenges—such as low event counts and spatial correlation due to shared local conditions.^6,7^

Difference-in-differences (DID) is widely used to address time-invariant unobserved confounding in program evaluations.^8^ However, it is usually applied at broader spatial levels—such as states or regions—or at the individual level, where spatial correlation is often negligible. At finer spatial scales, such as neighborhoods or grid cells, this assumption becomes less tenable, as unobserved spatially correlated factors may bias both effect and uncertainty estimates.

Bayesian disease-mapping models place spatially structured priors on area-level effects to account for spatial autocorrelation and produce more precise estimates by borrowing strength from neighboring areas.^9–12^ Although widely used for surveillance, they are rarely applied to DID studies. One reason may be that DID generally relies on two-way fixed effects to adjust for unobserved, time-invariant confounding, while disease-mapping models typically use random effects to model spatial dependence, which requires stronger assumptions for causal inference.^13^ However, recent studies show that a two-way Mundlak estimator—described in detail below—can recover the same causal estimates as fixed effects models while allowing residual dependence to be modeled using random effects.^14^

Meanwhile, new insights into DID methodology have revealed additional challenges when treatment timing varies across units (staggered adoption). In such settings, standard two-way fixed effects estimators may suffer from so-called forbidden comparisons, where already-treated units inappropriately serve as controls for later-treated ones.^15^ Several solutions to this problem have since been proposed,^14,16,17^ including imputation-based methods.^18^

Bayesian analysis provides a unified framework for counterfactual imputation and parameter estimation.^19^ Although Bayesian disease-mapping models are widely used in spatial epidemiology, we are not aware of prior methodologic work integrating them with imputation-based DID designs. To our knowledge, related literature has mainly focused on frequentist approaches to estimate treatment spillover effects (i.e., when interventions affect nearby untreated areas).^20–24^

In this article, we combine imputation-based DID methods with Bayesian disease-mapping models to improve precision and interval estimation in spatially correlated panel data, including settings with staggered adoption. By leveraging the equivalence between two-way Mundlak estimators and fixed-effects models,^14^ our approach maintains causal identification under standard DID assumptions while allowing residual structures to be modeled flexibly through structured random effects. We assess performance through simulations and apply the approach to evaluate ice cleat distribution programs in Swedish municipalities.

## SPATIAL IMPUTATION-BASED DID

We consider a balanced panel data setting where outcomes $Y_{it}$ are observed for i=1,2,..,N spatial units (areas) over t=1,2,…,T time periods. A subset of units ($N_{1}<N$) become treated during the observed period and remain treated thereafter, while the rest serve as never-treated controls. Treatment adoption may be simultaneous or staggered, but all areas are assumed to have at least one pretreatment period. Let $A_{it}$ be a binary indicator equal to 1 for postintervention observations in treated areas, and 0 otherwise. We use the term “treatment” following standard causal inference terminology, though it may refer to exposures, interventions, policies, etc.

Additionally, let $A_{i}={\left(A_{i1},A_{i2},\ldots A_{iT}\right)}^{\prime }$ denote the treatment vector for unit *i*, and $A=\left\{A_{1},\ldots ,A_{N}\right\}$ be the resulting N×T matrix of treatment assignments. Observed covariates are denoted by $X=\left\{X_{i1},\ldots ,X_{iT}\right\}$, which may vary over time or be time-invariant, though all included covariates are assumed unaffected by treatment to avoid bias from conditioning on intervention effects (i.e., to avoid collider bias^25^).

We denote potential outcomes as $Y_{it}\left(a\right)$, which is the outcome that would be observed if unit i were exposed to treatment level a∈{0,1} at time t. For simplicity, we adopt a standard consistency assumption:^26^

**Assumption 1 (consistency).**


$$\begin{array}{l} Y_{it}=A_{it}Y_{it}\left(1\right)+(1-A_{it})Y_{it}\left(0\right). \end{array}$$ (Eq. 1)

This assumption rules out treatment spillovers across units or over time—that is, potential outcomes are assumed to depend only on a unit’s own treatment status. While potentially strong for some small-area interventions (e.g., if hotspot policing in area i displaces crime to area j), it can be relaxed by redefining potential outcomes to depend on neighboring units’ treatment statuses. In such cases, $A_{it}$ may be reinterpreted as indicating direct or indirect exposure.^22^ We do not adopt this more general notation here, but estimation would proceed analogously, using only observations unexposed to either direct or spillover effects as controls.

Our goal is to estimate observation-level treatment effects $\tau_{it}=Y_{it}\left(1\right)-Y_{it}\left(0\right)$ for the treated set of observations ($\Omega_{1}=\left\{it:A_{it}=1\right\}$), and averages over subsets (e.g., the sample average treatment effect on the treated [SATT]). Under Assumption 1, we observe $Y_{it}=Y_{it}\left(1\right)$ for all treated observations, so identification only requires imputing $Y_{it}\left(0\right)$. To do this, we rely on data from the untreated set of observations ($\Omega_{0}=\left\{it:A_{it}=0\right\}$), consisting of both pretreatment periods for treated units and observations from never-treated controls.

This logic underlies the imputation-based DID estimator proposed by Borusyak et al.,^18^ which fits a model to the untreated data ($\Omega_{0}$) to impute the missing counterfactuals $Y_{it}\left(0\right)$ for treated observations ($\Omega_{1}$). A key advantage is that it avoids the forbidden comparisons problem outlined in the *Introduction*, which can arise under staggered adoption when models are fit to the full dataset. While alternative approaches address this by estimating and aggregating separate cohort-by-time contrasts,^16,17^ the imputation-based strategy aligns more naturally with Bayesian principles, which treat missing outcomes and model parameters jointly as random variables.^27^ It also supports spatiotemporal modeling without requiring a separate model for each cohort–time cell, which can fragment the data and disrupt continuity in spatial and temporal structures.

### A Spatiotemporal Model for the Untreated Potential Outcomes

To extend the imputation-based DID estimator to Bayesian spatiotemporal models, we begin by specifying a general model for the untreated potential outcomes $Y_{it}\left(0\right)$ that includes spatiotemporal dependence. Specifically, we impose the following restriction on the untreated outcomes:

**Assumption 2 (functional form).**


$$\begin{array}{l} Y_{it}\left(0\right)=\beta_{0}+X_{it}\beta +u_{it}, \end{array}$$ (Eq. 2)

where $\beta_{0}+X_{it}\beta$ represents the model’s fixed component, including an intercept $\beta_{0}$ and a vector of fixed coefficients β on observed covariates $X_{it}$. This component captures systematic variation and adjusts for confounding by observed variables. The term $u_{it}$ is a zero-mean residual that accounts for remaining variation, which we further decompose into:


$$\begin{array}{l} Y_{it}\left(0\right)=\beta_{0}+X_{it}\beta +\underset{u_{it}}{\underbrace{\alpha_{i}+\gamma_{t}+\Gamma_{it}+\epsilon_{it}}}, \end{array}$$ (Eq. 3)

where $\alpha_{i}$ captures time-invariant area effects, $\gamma_{t}$ denotes common time effects, $\Gamma_{it}$ represents spatiotemporal interactions, and $\epsilon_{it}$ is an independent and identically distributed error term.

In the Bayesian disease-mapping framework, each of these components may exhibit structured dependence; $\alpha_{i}$ may exhibit spatial autocorrelation, while $\gamma_{t}$ may follow some autoregressive or random-walk process. In practice, these terms are often further decomposed into structured and unstructured components—for example, $\alpha_{i}=\alpha_{i}^{s}+\alpha_{i}^{u}$, where $\alpha_{i}^{s}$ captures spatially structured variation (e.g., similarity across neighboring areas), and $\alpha_{i}^{u}$ captures independent excess variation.^9^ Similarly, $\Gamma_{it}$ represents spatiotemporal interactions,^10^ which may arise from processes such as infectious disease spread, shared weather conditions, or geographically clustered policy changes. Extensions of $u_{it}$ could also include spatially varying coefficients on covariates or area-specific trends.

Assumption 2 imposes a linear functional form consistent with conventional DID estimators but can be extended to generalized linear models. Since we model only untreated outcomes, treatment effect heterogeneity in $\tau_{it}$ remains unrestricted. To see this, note that combining Equations 1 and 3 gives:


$$\begin{array}{l} \begin{array}{llll} & Y_{it}=A_{it}Y_{it}\left(1\right)+(1-A_{it})Y_{it}\left(0\right) & \;\;\;\;=Y_{it}\left(0\right)+A_{it}\tau_{it} & \;\;\;\;=\underset{Y_{it}\left(0\right)}{\underbrace{\beta_{0}+X_{it}\beta +\alpha_{i}+\gamma_{t}+\Gamma_{it}+\epsilon_{it}}}+A_{it}\tau_{it}, \end{array} \end{array}$$ (Eq. 4)

which is a model with observation-specific treatment effects. The imputation framework, therefore, supports estimating both unit-level and aggregated effects (e.g., SATT) from a single model. When treatment intensity ($Z_{it})$ varies continuously (e.g., varying dose or participation), effects per unit of intensity can also be estimated using ${\hat{\tau }}_{it}/Z_{it}$, which allows for extension to (some) nonbinary treatments.^18^

### Causal Identification

Causal interpretation of DID estimates relies on the parallel trends assumption—that, absent treatment, both groups would have followed the same average outcome trajectory.^28^ This assumption extends to two-way fixed effects models, which generalize DID to settings with multiple periods and staggered or simultaneous treatment by estimating panel regressions with unit ($\mu_{i}$) and time ($\lambda_{t}$) fixed effects, which control for time-invariant unit differences and common period shocks, respectively.^12^

In quasi-experimental settings, Bayesian causal inference often relies on latent ignorability:

**Assumption 3 (latent ignorability).**


$$\begin{array}{l} \mathrm{Pr}\left(A_{i}|X_{i},Y_{i}\left(0\right),U_{i}\right)=\mathrm{Pr}(A_{i}|X_{i},U_{i}), \end{array}$$ (Eq. 5)

where $U_{i}=\left\{u_{i1},\ldots ,u_{iT}\right\}$ is a vector of unobserved latent variables.

This assumption generalizes standard unconfoundedness by allowing for unobserved confounding, provided it can be modeled by exploiting various features of the data. Parallel trends can be seen as a special case, where $u_{it}$ decomposes additively as $u_{it}=\mu_{i}+\lambda_{t}$.^19^

### Preserving Causal Identification in Random Effects Models

Modeling unit and time effects as fixed ensures causal identification under the parallel trends assumption, but prevents the use of random effects to capture spatial or temporal structure. Random effects, by contrast, allow for flexible modeling of residual dependence but typically assume no correlation with treatment—a strong and often unrealistic assumption that can bias effect estimates.

To address this problem, we adopt a two-way Mundlak estimator, which preserves the causal identification of fixed effects models while enabling the inclusion of structured random effects.^14^ This approach uses linear projections to decompose unit and time effects into components correlated with treatment and components orthogonal to it:


$$\begin{array}{l} \mu_{i}=\beta_{1}{\bar{A}}_{i}+\alpha_{i},\;and\;\lambda_{t}=\beta_{2}{\bar{A}}_{t}+\gamma_{t}, \end{array}$$ (Eq. 6)

where ${\bar{A}}_{i}$ proportion of time that unit *i* is treated and ${\bar{A}}_{t}$ is the proportion of areas treated at time *t*. The residuals $\alpha_{i}$ and $\gamma_{t}$ are, by construction, uncorrelated with the treatment indicator. If ${\bar{A}}_{i}$ and ${\bar{A}}_{t}$ are included as fixed covariates in the model, the remaining heterogeneity $\alpha_{i}$ and $\gamma_{t}$ can therefore be safely modeled using spatial and temporal random effects without sacrificing causal identification. The resulting model for untreated outcomes then becomes:


$$\begin{array}{l} Y_{it}\left(0\right)=\beta_{0}+\beta_{1}{\bar{A}}_{i}+\beta_{2}{\bar{A}}_{t}+X_{it}^{\prime }\beta^{\prime }+\alpha_{i}+\gamma_{t}+\Gamma_{it}+\epsilon_{it}, \end{array}$$   (Eq. 7)

where $X_{it}^{\prime }$ represents other covariates included in the fixed component aside from the Mundlak terms (Models including such terms are sometimes called hybrid, within-between, or correlated random effects models.^13^).

### Relaxing the Parallel Trend Assumption

Parallel trends rule out settings with treatment-correlated differential trends (i.e., time-varying confounding). Common strategies to relax this assumption include using unit- or group-specific pretreatment time trends, time-varying covariates (if unaffected by treatment), or interactive fixed effects (e.g., generalized synthetic controls^29^).

These approaches extend naturally to disease-mapping models. However, if treatment-correlated latent trends are modeled using random effects—such as unit-specific slopes—a corresponding Mundlak-type adjustment must be included, which can be derived via linear projection. For example, to adjust for unit-specific linear pretreatment trends, $\omega_{i}t$, we decompose:


$$\begin{array}{l} \omega_{i}t=\delta {\bar{A}}_{i}t+\kappa_{i}t \end{array}$$ (Eq. 8)

and add $\delta {\bar{A}}_{i}t$ to the fixed part of the model, allowing the residuals $\kappa_{i}t$—now orthogonal to treatment—to be modeled using (spatial) random effects. This generalizes to any time function g(t), including polynomial trends and interactive fixed effects (see eAppendix for details; https://links.lww.com/EDE/C272).

## IMPLEMENTATION USING INTEGRATED NESTED LAPLACE APPROXIMATION

Disease-mapping models can be estimated using conventional Bayesian analysis methods.^12^ In this article, we implement the approach using integrated nested Laplace approximation,^30,31^ which offers a fast, deterministic alternative for disease-mapping models with Gaussian random effects.^32^ We focus on this method due to its computational efficiency and ease of use, although Markov Chain Monte Carlo methods may be preferable for more complex models (e.g., with non-Gaussian effects).

Disease-mapping models can be fit using the R-INLA package.^31^ Implementation of the proposed framework only differs from standard disease-mapping applications in that we fit the model only to untreated observations ($\Omega_{0}$). This can be done by setting postintervention outcomes in treated units ($\Omega_{1}$) to missing before estimating the model. R-INLA then imputes ${\hat{Y}}_{it}\left(0\right)$ for these observations using parameters estimated from $\Omega_{0}$. Observation-level treatment effect estimates are then computed as:


$$\begin{array}{l} {\hat{\tau }}_{it}=Y_{it}-{\hat{Y}}_{it}\left(0\right), \end{array}$$ (Eq. 9)

and can be aggregated as needed (e.g., averaged over all postintervention treated observations to estimate the SATT).

### Posterior Predictive Inference

Bayesian inference assigns prior distributions to model parameters and updates them via Bayes’ theorem to obtain posterior distributions given the observed data and model. Predicting unobserved outcomes for treated observations in $\Omega_{1}$ involves posterior predictive inference, which directly quantifies uncertainty in counterfactuals and treatment effects.^33^ This is done by sampling counterfactual outcomes ${\hat{Y}}_{it}\left(0\right)$ from the joint posterior distribution of the model’s fitted values (obtained via the inverse link function, unless the identity link is used) and any likelihood-specific nuisance parameters. In R-INLA, this can be implemented using *inla.posterior.sample*(), which returns draws of the linear predictor and likelihood parameters. To obtain posterior predictive outcomes, these must be combined. For example, under a Gaussian likelihood, counterfactuals are drawn as


$$\begin{array}{l} {\hat{Y}}_{it}^{\left(s\right)}\left(0\right)∼N\left(\eta_{it}^{\left(s\right)},\sigma_{\epsilon }^{2}\right), \end{array}$$ (Eq. 10)

where $\eta_{it}^{\left(s\right)}$ is the fitted value for draw s, based on all model components except the error term $\epsilon_{it}$, and $\sigma_{\epsilon }^{2}$ is the observation-level variance. The draw-specific treatment effect is


$$\begin{array}{l} {\hat{\tau }}_{it}^{\left(s\right)}=Y_{it}-{\hat{Y}}_{it}^{\left(s\right)}\left(0\right), \end{array}$$ (Eq. 11)

and (draw-specific) average effects are obtained by averaging ${\hat{\tau }}_{it}^{\left(s\right)}$ over relevant $\left(i,t\right)\in \Omega_{1}$ within each draw. Credible intervals are then constructed from percentiles of the resulting posterior distribution (e.g., 2.5th and 97.5th percentiles for a 95% interval). This approach yields valid inference under Assumptions 1–3,^19^ provided standard conditions on treatment assignment and positivity hold (see eAppendix; https://links.lww.com/EDE/C272 for details).

### Spatial and Temporal Structures

Integrated Nested Laplace Approximation fits Bayesian hierarchical models whose latent effects are represented as Gaussian Markov random fields. Each latent component is defined by a structure matrix that captures dependence and an accompanying scalar precision parameter that is estimated from the data.^30^ With an identity structure matrix, the field reduces to exchangeable random effects, whereas a neighbor-based matrix—typically defined by shared borders or distance thresholds—induces spatial dependence.^11^

For areal panel data, a common choice is the intrinsic conditional autoregressive prior, which pools information conditionally across neighboring areas.^11,34^ The widely used Besag–York–Mollié model augments this prior by adding an unstructured effect to capture excess local heterogeneity.^9^ A data-adaptive mixing parameter ϕ∈[0,1] can also be added to govern the share of spatial versus unstructured variance (ϕ=0 gives a pure unstructured model, ϕ=1 a simple intrinsic conditional autoregressive model).^35^ Alternatives also exist that allow for adaptive (localized) smoothing.^36,37^

Temporal dependence can be added through discrete-time structures such as autoregressive and random-walk specifications. Integrated Nested Laplace Approximation also facilitates spatiotemporal interaction models by combining spatial and temporal precision matrices via Kronecker products.^10,38^

### When Can Precision Gains be Expected?

Precision gains from spatial smoothing arise when nearby areas truly are similar. They are greatest when outcomes are noisy (e.g., small populations or rare events), spatial dependence is strong, and each area has many genuinely similar neighbors.^9,35,36^ When neighboring areas differ sharply or the neighbor matrix is poorly specified, smoothing yields little benefit and may even introduce bias.^39^

### Model Specification

Two-way fixed effects models leave unit and time effects unrestricted, whereas disease-mapping requires explicit assumptions about the structure and distribution of random effects.^32,38^ This trade-off is important, as precision gains and interval estimates depend on correct model specification. Integrated Nested Laplace Approximation fits models where random effects are assumed to be approximately Gaussian.^40^ Model fit can be assessed using tools, such as the deviance information criterion or cross-validation.^41^ Extensions to non-Gaussian random effects are also possible, for example, through postestimation tools like the *ngvb* package in R.^42^

Bayesian inference requires specifying priors for all model parameters. In R-INLA, default priors for fixed effects are typically vague, allowing estimates to be largely driven by the data, while penalized complexity priors are increasingly used for random effects.^38,43^ Penalized complexity priors shrink random effects toward a zero-mean constant unless strongly supported by the data, which essentially removes them from the model. For example, one can specify penalized complexity priors on all random effects in Equation 7 to allow the model to simplify to a nonspatial two-way Mundlak regression when there is limited evidence of spatiotemporal dependence.

## Simulation Study

We conducted a simulation study to assess the proposed method compared to conventional DID. Specifically, we assessed if it (1) is unbiased when causal assumptions hold, including in settings with heterogeneous effects and staggered adoption; (2) improves precision and interval coverage under spatiotemporal dependence; and (3) achieves comparable precision and coverage in nonspatial data.

Main results are summarized below; technical details and full results are provided in the eAppendix; https://links.lww.com/EDE/C272.

### Design

We simulated panel data with and without spatiotemporal dependence under three adoption settings: (1) simultaneous adoption with homogeneous effects, (2) simultaneous adoption with heterogeneous effects, and (3) staggered adoption with heterogeneous effects. We evaluated performance across three data scenarios: no spatiotemporal dependence with unconditional parallel trends (Scenario 1), spatiotemporal dependence with unconditional parallel trends (Scenario 2), and spatiotemporal dependence with parallel trends conditional on area-specific linear trends (Scenario 3).

We induced dependence using intrinsic conditional autoregressive priors for spatial effects, first-order autoregressive terms for temporal effects, and interactions between these. Each simulation included 50 areas, with 10 treated either midway or, in the staggered case, at randomly selected time points thereafter. We introduced confounding by correlating treatment probabilities with area effects and, in Scenario 3, also with area-specific trends. In settings with homogeneous effects, we set $\tau_{it}=-5$ for all treated areas. In heterogeneous settings, we added standard Gaussian noise around −5 and a linear slope change at the time of the treatment, with area-specific coefficients drawn from a uniform distribution between −0.25 and −0.75.

We report results for T=10; findings were similar for shorter (T=6) and longer (T=20) panels (see eAppendix; https://links.lww.com/EDE/C272). We compare the proposed two-way Mundlak approach with a conventional frequentist DID estimator with area-level cluster-robust standard errors applied to the full dataset. We also benchmark against disease-mapping models without two-way Mundlak adjustments. In each scenario, we fit a spatiotemporal model with either correctly specified or intentionally overspecified dependence structures. In Scenario 1, we deliberately overspecified the model to test whether the penalization would effectively shrink unnecessary spatial terms in the absence of structured dependence. In Scenario 3, we included area-specific trends in both approaches; in spatial models, we allowed for spatially smoothed trends. We assessed performance using bias, root mean squared error (RMSE), and 95% interval coverage for the SATT.

### Summary of Simulation Results

**FIGURE 1.**
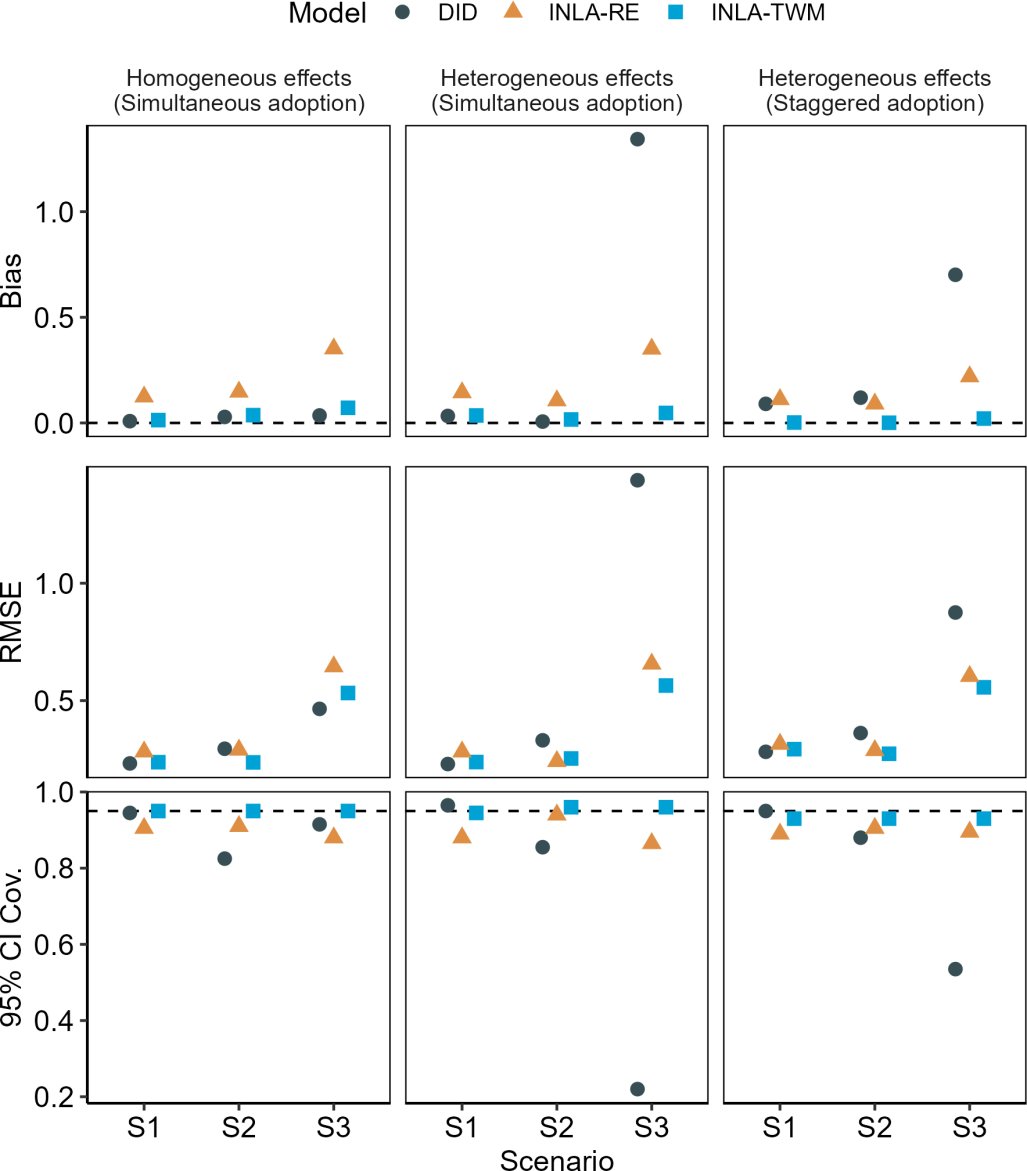
Simulation results by scenario and adoption setting comparing frequentist difference-in-differences (DID) estimated using two-way fixed effects models on the entire dataset (with cluster-robust standard errors) and imputation-based DID using disease-mapping models estimated via INLA, with (“INLA-TWM”) and without (“INLA-RE”) two-way Mundlak adjustments. Scenario 1 (S1) assumes no spatiotemporal dependence, with unconditional parallel trends. Scenario 2 (S2) introduces residual dependence via an intrinsic conditional autoregressive spatial structure, first-order autoregressive temporal effects, and a spatiotemporal interaction. Scenario 3 (S3) adds spatially correlated linear trends to S2, inducing nonparallel trends bias; models in S3 include area-specific trends. Results are based on 200 simulations. Reported metrics include bias, root mean squared error (RMSE), and 95% credible/confidence interval (CI) coverage for the sample average treatment effect on the treated. Horizontal lines indicate target values where applicable: 0 for bias, 0.95 for CI coverage; for RMSE, lower values are better, though no universal benchmark applies.

Figure 1 shows results by scenario and treatment adoption setting. As expected, the Integrated Nested Laplace Approximation model without Mundlak terms exhibited bias across all model settings and scenarios, which confirms that the Mundlak adjustments are needed to ensure causal identification. In Scenario 1 (no residual structure), DID and the proposed approach (with two-way Mundlak terms) performed similarly under simultaneous adoption (Columns 1–2), indicating that the model’s penalization scheme behaves as intended. Under staggered adoption with heterogeneous effects (Column 3), DID showed bias from forbidden comparisons, while the proposed approach remained unbiased.

In Scenario 2 (with spatiotemporal dependence), the proposed approach outperformed DID on precision (lower RMSE) and interval coverage, with similar bias. These patterns mostly held in Scenario 3 (area-specific nonparallel trends), with two exceptions. Under homogeneous effects (Column 1), DID showed slightly better precision, likely due to full-data estimation of area-specific trends. Under heterogeneous effects (Columns 2–3), DID exhibited substantial bias as trends estimated from the full data partially absorbed the intervention trend change effect. In contrast, the proposed approach remained unbiased because it only relies on untreated observations for estimation.

### Supplementary Analyses and Results

Additional results are available in the eAppendix; https://links.lww.com/EDE/C272. We obtain similar results for settings with only a single treated unit and for estimates of area-specific average postintervention effects. However, when spatiotemporal dependence ($\Gamma_{it}$) is present, we also find that these estimates become less accurate for areas with few untreated neighbors. This occurs because the spatial terms in our simulations (and commonly used in the literature) use first-order neighbors to estimate spatial structures; if untreated potential outcomes are missing for all neighboring areas, estimation accuracy decreases. In practice, these estimates should therefore be interpreted with caution.

## EMPIRICAL EXAMPLE: ICE CLEAT DISTRIBUTION

We illustrate the method by re-analyzing data from Eklund et al.,^44^ who used an imputation-based DID estimator to evaluate the impact of ice cleat distribution programs on ice-related fall injuries in Swedish municipalities. Ethical approval was obtained from the Swedish Ethical Review Authority (diary no. 2021-10338).

### Data

**FIGURE 2.**
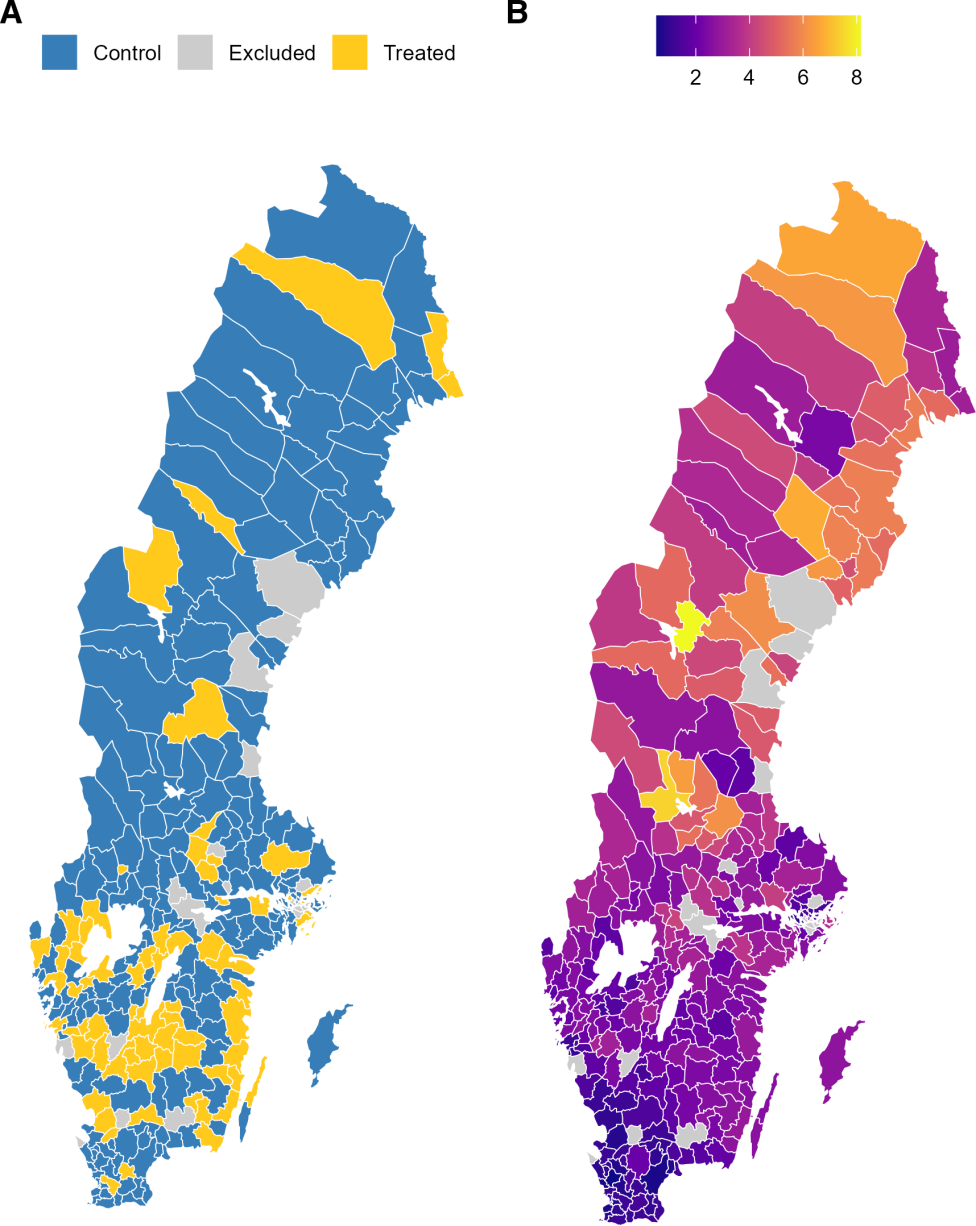
Panel A, Swedish municipalities with (n = 73) and without (n = 200) ice cleat distribution programs (per survey data collected in 2019; n = 17 municipalities with known programs but lacking survey data on intervention start date and/or clear age thresholds excluded). Panel B, Average number of patients treated for injuries after falls on snow or ice in specialized outpatient or inpatient care per 1,000 person-winters from the winter 2001/2002 to 2018/2019, according to data from the National Patient Register.

The dataset includes 273 municipalities observed over 17 winter periods (October 1 to April 30, from 2001/2002 to 2018/2019). Ice cleats were distributed in 73 municipalities (Figure 2A), with staggered adoption and an average follow-up of 3.5 winters postintervention.

Implementation varied across municipalities but primarily targeted citizens aged 65 and older (85% of treated municipalities), with higher age thresholds used in the rest. Distribution occurred either at municipality-owned buildings (e.g., libraries) or through mailed coupons redeemable at local stores. Survey and repeated cross-sectional data indicate that approximately 40% of eligible citizens collected cleats, with self-reported use increasing by 7.5 percentage points in eligible age groups and no discernible change in ineligible groups.^45,46^

Outcomes were defined as ice-related fall injury rates per 1,000 eligible citizens per winter. In treated municipalities, eligibility was based on the program-specific age threshold; in control municipalities, the threshold was set to the most common criterion, age 65 and older.

### Modeling Strategy

Figure 2B shows the spatial outcome distribution of outcomes. Spatial and spatiotemporal variation are expected given regional climate differences and local year-to-year weather shocks that affect neighboring municipalities similarly. We account for these dependencies using spatiotemporal models with various specifications detailed in Table. We also included injury rates in age groups 1–15 years below the eligibility threshold as a time-varying covariate with both fixed and spatially varying effects to adjust for local time-varying shocks affecting all age groups—analogous to the triple differences specification in Eklund et al.^44^

**TABLE.** Estimated Average Effects of Ice Cleat Distribution Programs in 73 Swedish Municipalities on Ice-related Fall Injury Rates (per 1,000 person-winters) Among Older Adults, Using Unstructured Frequentist and Bayesian Spatial Imputation-Difference-in-Differences (DID) Models

	Frequentist DID	Bayesian spatial DID		
	Original	Model 1	Model 2	Model 3
Estimate (95% CI)	−0.24 (−0.49, 0.02)	−0.23 (−0.51,0.03)	−0.17 (−0.40, 0.06)	−0.21 (−0.45, 0.01)
95% CI width	0.51	0.54	0.47	0.46
Ineligible age controls	Yes	No	Yes	Yes
Spatiotemporal interactions	No	No	No	Yes
Deviance information criterion		10197.7	8781.2	8457.3
Spatial variance contribution (ϕ)		0.61	0.91	0.99

Notes: Estimates represent sample average treatment effects on ice-related injury rates per 1,000 person-winters across all treated municipalities and post-intervention periods. 95% confidence or credible intervals (CI) are shown in parentheses. Models with ineligible age controls use injury rates from age groups 1–15 years below the eligibility threshold as controls. All Bayesian spatial DID models are estimated using a Gaussian likelihood and include two-way Mundlak terms, first-order autoregressive global temporal random effects, and Besag–York-Mollié spatial effects (parameterized following Riebler et al.^35^). Model 2 includes injury rates among ineligible age groups as a time-varying covariate with both fixed and spatially varying coefficients. Model 3 further includes a spatiotemporal interaction between a first-order autoregressive temporal effect and an intrinsic conditional autoregressive spatial effect. Penalized complexity priors (with R-INLA default values) are applied to the precision of all random effects, and each random effect is constrained to sum to zero to improve model identifiability. Credible intervals are based on 1,000 posterior predictive draws. The spatial variance contribution refers to the mixing parameter in Riebler et al.’s re-parameterized Besag–York-Mollié model. The frequentist DID model is a triple differences specification with ineligible age controls from Eklund et al.,^44^ estimated using the imputation-based DID approach by Borusyak et al.^18^

## RESULTS

The original estimate suggested a reduction in injury rates of −0.24 per 1,000 person-winters, but the 95% confidence interval marginally overlapped zero (−0.49, 0.02), leaving open the possibility of no effect or a slight risk increase (Table).^44^

We compare this with three disease-mapping models. A basic spatial model without ineligible age controls and spatiotemporal interactions (Model 1) produced similar results but with a slightly wider interval, likely due to omitting the ineligible age controls. Adding these (Model 2) and then including spatiotemporal interactions (Model 3) improved model fit and precision (Table).

All models indicate strong spatial dependence (Table). Model 3 had the best fit according to the deviance information criterion. Its estimate (−0.21, 95% CI = −0.45, 0.01) reduced the interval width by about 10%, but also shifted the estimated effect slightly toward the null, so conclusions remain the same. This shift does not suggest bias; rather, it reflects how spatial smoothing can yield more stable estimates when autocorrelation is present and the model better captures underlying structure.

## DISCUSSION

Spatial dependence and noisy outcomes are common in epidemiologic data, particularly with small-area units, but these problems are often overlooked in program evaluations using DID designs. In this article, we have considered how Bayesian disease-mapping models can be integrated into an imputation-based DID framework to account for spatial dependence and improve estimation precision. We find that when combined with a two-way Mundlak estimator, these models can improve precision and uncertainty estimation while preserving the identification strategy of conventional DID—even with staggered adoption and heterogeneous effects—provided the spatiotemporal structure is correctly specified or conservatively overspecified to allow penalization of unsupported terms.

Overall, our results support the idea that disease-mapping models can be useful for causal panel data analyses with spatially correlated data, but there are many possible ways to develop these ideas further. For example, formally extending the framework to incorporate spatial synthetic controls would be valuable to help relax the parallel trend assumption (we outline a simple two-step implementation using Integrated Nested Laplace Approximation in the eAppendix; https://links.lww.com/EDE/C272 but a fully Bayesian version would likely require Markov Chain Monte Carlo estimation).^29^ A related contribution by Vega and Nethery^47^ also proposes a Bayesian spatiotemporal matrix completion method—conceptually similar to synthetic controls—for modeling rare disease incidence with spatial and temporal priors. Wooldridge discusses extensions of the two-way Mundlak estimator to Poisson and logistic models.^14^ In principle, our framework could be applied in these settings, as Integrated Nested Laplace Approximation supports generalized linear models, but Mundlak-type adjustments may be less efficient in nonlinear models.^48^ Assessing performance in nonlinear models is therefore an important direction for future research. Studies investigating more complex data settings, including continuous treatments, treatment spillover effects, multiple outcomes, and coordinate data, would also be valuable.

## ACKNOWLEDGMENTS

We are grateful for constructive feedback provided by Niklas Jakobsson (Karlstad University) and Mikael Svensson (University of Gothenburg) on earlier drafts of this paper.

## Supplementary Material


