# Supplementary material for: Spatial Difference-in-Differences with Bayesian Disease Mapping Models
Source: Epidemiology. 2025 Sep 10;37(1):30–8. doi: 10.1097/EDE.0000000000001912 (PMC12643553; doi:10.1097/EDE.0000000000001912)
Supplement: Supplementary file 1 [file ede-37-030-s001.pdf]

eAppendix for
“Spatial Difference-in-Differences with Bayesian Disease Mapping Models”

Carl Bonander[1,2*], Marta Blangiardo[3], and Ulf Strömberg[1]

[1]School of Public Health & Community Medicine, University of Gothenburg, Sweden

[2]Center for Societal Risk Research, Karlstad University, Karlstad, Sweden

[3]MRC Centre for Environment and Health, Department of Epidemiology & Biostatistics, School of Public Health, Imperial College London, London, UK

*Correspondence: carl.bonander@gu.se.

Contents

A Assumptions on the treatment assignment mechanism **3**

B Simulation study: methods and full results **4**

B.1 Spatial and temporal dependence structures 5

B.2 Treatment assignment 5

B.3 Effects and realized outcomes 6

B.4 Scenarios 7

B.5 Simulation results 8

C Latent factor models **13**

D Tables **16**

A Assumptions on the treatment assignment mechanism

As discussed briefly in Section 3, to formally motivate Bayesian inference in our setting, we must impose a probabilistic model on the treatment assignment mechanism, $\Pr(A \mid X, Y(0))$ (Pang et al. 2022). Specifically, we assume:

Assumption A1 (Individualistic Assignment and Positivity).

$$\Pr(A \mid X, Y(0)) = \prod_{i=1}^{N} \Pr(A_i \mid X_i, Y_i(0)), \quad \text{with} \quad 0 < \Pr(A_i \mid X_i, Y_i(0)) < 1 \quad \text{for all } i.$$

The first part of this assumption—*individualistic assignment*—states that the treatment assignment in area i depends only on its own covariates and potential outcomes, and not on those of other areas. This rules out settings where area i adopts treatment simply because another area j does, for reasons unrelated to area i's own characteristics X_i and potential outcomes $Y_i(0)$. Treatment assignment may still exhibit spatial correlation, provided such dependence operates through X_i and $Y_i(0)$.

The second part—*positivity*—requires that all areas have a non-zero probability of receiving treatment. This is needed to justify the use of untreated observations to impute counterfactual outcomes for treated observations.

B Simulation study: methods and full results

We conduct a series of Monte Carlo experiments to evaluate the performance of our proposed method under different data-generating scenarios with and without spatiotemporal dependence structures. Code to reproduce the simulations are also available at Open Science Framework.[1]

We simulate data across various scenarios to evaluate the performance of the proposed method. The data-generating process (DGP) in each scenario starts with a baseline model for the untreated potential outcomes, $Y_{it}(0)$, that incorporates various dependence structures. Untreated potential outcomes follow the general form:

$$Y_{it}(0) = \beta_0 + \boldsymbol{X}\beta + u_{it}, \tag{1}$$

where $\beta_0 + \boldsymbol{X}\beta$ represents a fixed component, including an intercept (β_0) and a vector of fixed coefficients (β) on observed covariates ($\boldsymbol{X}$). The term u_{it} captures unexplained variation and is further expanded as:

$$Y_{it}(0) = \beta_0 + \boldsymbol{X}\beta + \alpha_i + \gamma_t + \Gamma_{it} + \epsilon_{it}, \tag{2}$$

where α_i represents time-invariant area effects, γ_t captures common time effects, Γ_{it} are spatiotemporal interactions, and ϵ_{it} denotes i.i.d. observation-level errors. Each effect, except for the random errors, may be structured or unstructured as described in the main paper. The model may also be expanded to include random coefficients on covariates. In Scenario 3 of the simulations, we add a spatially structured linear time trend, $\omega_i \times t$, where ω_i is the area-specific slope.

Structured effects are generated using the `inla.qsample` function in R-INLA (Lindgren and Rue 2015), which samples from a Gaussian Markov Random Field (GMRF) (Rue and Held 2005). This function requires a precision matrix $\boldsymbol{Q}$, defined as $\boldsymbol{Q} = \kappa\boldsymbol{K}$, where κ is a scalar precision parameter (the inverse of variance, $\kappa = \frac{1}{\sigma^2}$)

[1] Link temporarily anonymized for peer-review.

and $\boldsymbol{K}$ is the structure matrix (Knorr-Held 2000). To simplify, we set $\kappa = 1$, making $\boldsymbol{Q} = \boldsymbol{K}$, which implies $\sigma^2 = 1$.

B.1 Spatial and temporal dependence structures

For spatial dependence, we use an intrinsic conditional autoregressive (ICAR) model (Besag 1974). The ICAR structure matrix, $\mathbf{K}_{\text{ICAR}}$, has non-diagonal elements $k_{ij} = -1$ if units i and j are contiguous, diagonal elements $k_{ii} = m_i$ (where m_i is the number of neighbors for unit i), and zeros elsewhere (Knorr-Held 2000). Throughout, we use a 5×10 lattice structure to define the neighbor matrix for $N = 50$ areas (Figure A1).

For temporal dependence, we apply an AR(1) structure with the following structure matrix:

$$\mathbf{K}_{\text{AR}(1)} = \begin{bmatrix} 1 & -\rho & 0 & \cdots & 0 \\ -\rho & 1+\rho^2 & -\rho & \cdots & 0 \\ 0 & -\rho & 1+\rho^2 & \cdots & 0 \\ \vdots & \vdots & \vdots & \ddots & \vdots \\ 0 & 0 & 0 & \cdots & 1 \end{bmatrix}, \tag{3}$$

with $\rho = 0.5$ throughout.

Structure matrices for spatiotemporal interactions, used to simulate Γ_{it}, are defined as $\mathbf{K}_{\text{AR}(1)} \otimes \mathbf{K}_{\text{ICAR}}$, where $\otimes$ is the Kronecker product (Knorr-Held 2000). Unstructured residuals, ϵ_{it}, are drawn from an i.i.d. standard Gaussian distribution throughout.

B.2 Treatment assignment

We simulate a treatment probability π_i for each area i using a logistic model

$$logit(\pi_i) = \alpha_i + \omega_i, \tag{4}$$

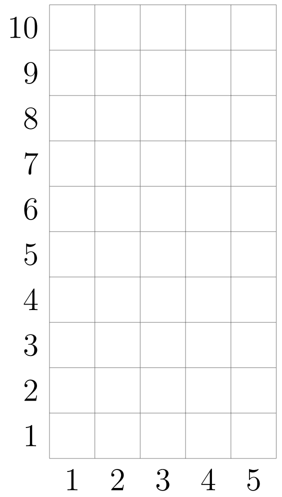

Figure A1: Lattice used as neighbor matrix. Units that share a border, including diagonally, are considered neighbors.

where ω_i is a unit-specific linear trend parameter set to zero in scenarios where the common trend assumption holds. We then select N_1 units for treatment with sampling probability π_i.

In settings with simultaneous adoption, all treated areas are treated at $T/2$. With staggered adoption, we randomly draw a treatment time c_i between $T/2$ and T for each treated area.

B.3 Effects and realized outcomes

We also simulate effects, $\tau_{it} = Y_{it}(1) - Y_{it}(0)$. For settings with homogeneous effects, we set $\tau_{it} = -5$ for all units and time points. With heterogeneous effects, we define $\tau_{it} = -5 + \alpha_i + A_{it}(t - c_i)\psi_i$, where A_{it} is an intervention dummy and ψ_i is a linear trend change effect drawn randomly from a uniform distribution between -0.75 and -0.25. Finally, we set the realized outcomes to

$$\begin{aligned} Y_{it} &= A_{it}Y_{it}(1) + (1 - A_{it})Y_{it}(0) \\ &= Y_{it}(0) + A_{it}\big(Y_{it}(1) - Y_{it}(0)\big) \qquad (5) \\ &= Y_{it}(0) + A_{it}\tau_{it}. \end{aligned}$$

For performance evaluation, we use two effect measures. The first is the *sample*

average treatment effect on the treated ($SATT$):

$$SATT = \frac{1}{\sum_{it} A_{it}} \sum_{it:A_{it}=1} \big(Y_{it}(1) - Y_{it}(0)\big), \tag{6}$$

where the summation is over all treated observations. The second is the *area-specific average treatment effect* ($SATT_i$):

$$SATT_i = \frac{1}{P_i} \sum_{t=c_i}^{T} \big(Y_{it}(1) - Y_{it}(0)\big), \tag{7}$$

where P_i is the number of post-intervention periods for unit i. For the corresponding estimators, we simply replace $Y_{it}(1) = Y_{it}$ (as follows from Equation 5) and $Y_{it}(0)$ with the estimated $\hat{Y}_{it}(0)$.

B.4 Scenarios

Below, we define each scenario in terms of the DGP for untreated potential outcomes $Y_{it}(0)$.

Scenario 1: Unstructured data

$$\begin{aligned}
\alpha_i &\sim \mathcal{N}(0, 1), \\
\gamma_t &\sim \mathcal{N}(0, 1), \\
\omega_i &= 0, \\
\epsilon_{it} &\sim \mathcal{N}(0, 0.25).
\end{aligned}$$

Scenario 2: Spatiotemporal dependence and common trends

$$\begin{aligned}
\alpha_i &\sim \text{ICAR}(\mathbf{Q}_{\text{ICAR}}), && \text{with } \mathbb{E}[\alpha_i] = 0,\ \text{SD} = 1,\\
\gamma_t &\sim \text{AR}(1)(\rho = 0.5), && \text{with } \mathbb{E}[\gamma_t] = 0,\ \text{SD} = 1,\\
\omega_i &= 0, &&\\
\Gamma_{it} &\sim \text{GMRF}(\mathbf{Q}_{\text{AR}(1)} \otimes \mathbf{Q}_{\text{ICAR}}), && \text{with } \mathbb{E}[\Gamma_{it}] = 0,\ \text{SD} = 1,\\
\epsilon_{it} &\sim \mathcal{N}(0, .25).
\end{aligned}$$

Scenario 3: Spatially varying trends

$$\begin{aligned}
&\mathbf{X} \text{ consists of a time variable } t = 1, 2, \ldots, T, &&\\
&\alpha_i \sim \text{ICAR}(\mathbf{Q}_{\text{ICAR}}), && \text{with } \mathbb{E}[\alpha_i] = 0,\ \text{SD} = 1,\\
&\gamma_t \sim \text{AR}(1)(\rho = 0.5), && \text{with } \mathbb{E}[\gamma_t] = 0,\ \text{SD} = 1,\\
&\omega_i \sim \text{ICAR}(\mathbf{Q}_{\text{ICAR}}), && \text{with } \mathbb{E}[\omega_i] = 0,\ \text{SD} = 0.5,\\
&\Gamma_{it} \sim \text{GMRF}(\mathbf{Q}_{\text{AR}(1)} \otimes \mathbf{Q}_{\text{ICAR}}), && \text{with } \mathbb{E}[\Gamma_{it}] = 0,\ \text{SD} = 1,\\
&\epsilon_{it} \sim \mathcal{N}(0, .25).
\end{aligned}$$

B.5 Simulation results

For each simulation, we collect metrics for the estimated SATT, including bias, root mean squared error (RMSE), 95% confidence/credible interval (CI) width, and 95% CI coverage, to measure implications for causal validity, precision, and statistical inference. The spatiotemporal model used in each scenario is either correctly specified or over-specified but includes mechanisms to penalize it toward the correct specification (as discussed in the main paper).

We compare three variants of the spatiotemporal model: (1) our preferred model, which includes the two Mundlak variables (TWM-INLA), (2) a version without the Mundlak variables (non-TWM-INLA), and (3) a model that replaces random effects for α_i (area effects) and γ_t (common time effects) with fixed effects but is otherwise

identical to the non-TWM model (TWFE-INLA). For additional benchmarks, we include ordinary least squares (OLS) with a simple treatment dummy, standard Frequentist DID, and Frequentist DID with linear unit-specific trends. Confidence intervals for the Frequentist models are based on cluster-robust standard errors clustered by area (Cameron and Miller 2015).

We run each scenario three times: (i) with homogeneous treatment effects under simultaneous adoption and (ii) with heterogeneous effects under simultaneous and (iii) staggered adoption. Heterogeneous effects include both a spatial area effect and an area-specific linear trend change.

The first set of simulations provides a fair comparison with standard DID approaches, which assume homogeneous effects unless explicitly modeled, allowing us to isolate the impact of accounting for spatial dependence. The second and third sets introduce (ii) effect heterogeneity and (iii) forbidden comparisons bias (Goodman-Bacon 2021), assessing more realistic scenarios where imputation-based methods should be preferable (Borusyak et al. 2024).

We generate data for $N = 50$ units, with $N_1 = 10$ treated at the midpoint $(T/2)$ of the observation period in simulations with simultaneous treatment adoption. For staggered adoption, treatment initiation occurs at a randomly drawn time point between $T/2$ and T. Time series lengths (T) vary between 6, 10, and 20, and we run 200 replications for each scenario and setting. Depending on context, these data represent either typical or relatively small cross-sectional sample sizes—large in analyses of U.S. states but small in small-area studies. The time series lengths range from short to long, particularly if the time interval represents years.

Scenario 1: Unstructured data In the first scenario, we test the method's data-driven model selection capability in the absence of spatial or temporal dependence. We generate data to (unconditionally) satisfy the parallel trends assumption, and no spatial or temporal structure is introduced. We fit an over-specified spatiotemporal model including a BYM2 area effect ($\alpha_i = \alpha_i^u + \alpha_i^s$), unstructured and AR(1) time effects ($\gamma_t = \gamma_t^u + \gamma_t^s$), and a spatiotemporal interaction modeled using an

AR(1)×ICAR process (Γ_{it}). Residual errors (ϵ_{it}) are modeled as i.i.d. Gaussian. We apply sum-to-zero constraints on random effects to improve model identifiability (Goicoa et al. 2018).

Under homogeneous effects with simultaneous adoption, the TWM-INLA and TWFE-INLA models perform comparably to standard Frequentist DID across all metrics despite including spatiotemporal random effects, indicating that the model selection strategy works as intended (Table A2, Scenario 1). Meanwhile, and as expected, the non-TWM-INLA model exhibits some bias, confirming that the spatiotemporal random effects cannot be trusted to handle all bias alone. When we introduce heterogeneous effects under simultaneous adoption, the imputation-based INLA models perform better than Frequentist DID in terms of inference and precision, though the regular DID model remains unbiased (Table A3, Scenario 1). However, the DID model with linear trends exhibits severe bias because the heterogeneous trend change effects are absorbed into the linear trend parameters when these are estimated on both pre-and-post-intervention data. Similar results appear in the scenario with staggered adoption, but the regular DID model exhibits bias due to forbidden comparisons (Goodman-Bacon 2021), even when the parallel trend assumption holds (Table A4, Scenario 1). As expected (Borusyak et al. 2024), the latter phenomenon is not present in the imputation-based INLA models.

Scenario 2: Spatiotemporal dependence and parallel trends In the second scenario, we introduce spatial and temporal dependence to evaluate the benefits of accounting for these features. Spatial dependence is generated using an ICAR structure applied to a 5×10 grid and temporal dependence is generated using an AR(1) structure with an autoregressive parameter of $\rho = 0.5$. Spatiotemporal interactions are also included. Treatment assignment is spatially correlated, based on unit-specific probabilities derived from the area effects with a logistic model. The parallel trends assumption remains satisfied, and we fit the same spatiotemporal models as in Scenario 1.

The results mirror those of the unstructured scenario in terms of bias patterns.

Notably, the spatiotemporal INLA models demonstrate better performance than Frequentist DID in terms of inference and precision across all settings (Tables A2–A4, Scenario 2). Moreover, while there are no clear differences in bias between TWM-INLA and the TWFE-INLA models, the TWM-INLA model shows superior 95% CI coverage compared to the TWFE-INLA model, likely because it can fully account for the spatiotemporal dependence in the time-variant area effects and common time effects.

Scenario 3: Spatially varying, non-parallel trends In the third scenario, we introduce spatially varying linear trends generated using an ICAR structure. Treatment assignment depends on both the area effects and these trends, violating the parallel trends assumption. The spatiotemporal models include all components from previous scenarios, with an additional BYM2 effect for unit-specific trends, a fixed global linear trend, and a linear trend $\times$ treatment group interaction in the fixed component.

With homogeneous effects and simultaneous adoption, the TWM-INLA models perform well in terms of bias and 95% CI coverage (Table A2, Scenario 3). However, the Frequentist DID model, which utilizes all data—including post-treatment observations—to estimate unit-specific trends, achieves higher precision. This is expected, as the imputation-based INLA models discard post-treatment observations to allow for effect heterogeneity. Nonetheless, if treatment effects are truly homogeneous, then using the full dataset can improve accuracy. When effects are heterogeneous, both with and without staggered adoption, the TWM-INLA model consistently outperforms the Frequentist alternative across all evaluation metrics (Tables A3–A4, Scenario 3).

Additional analyses We conduct additional analyses to evaluate specific use cases and robustness.

Frequentist DID models with conventional cluster-robust standard errors often perform poorly when the number of treated units is small (Conley and Taber 2011).

Bayesian posterior predictive inference, however, is not subject to the same biases (Pang et al. 2022). To verify if this is true also in the context of the proposed framework, we assess performance in a scenario with only a single treated unit ($N_1 = 1$). As expected, the Frequentist DID model performs poorly across all settings, whereas the Bayesian TWM-INLA approach achieves performance comparable to the scenarios in Tables A2–A4 (Table A5).

Our main analyses focus on the SATT averaged over ten treated units, but analysts may also be interested in area-specific effects—for instance, to examine spatial effect heterogeneity or identify areas where an intervention was particularly successful or ineffective. In Table A6, we present performance measures for area-specific effect estimates. The patterns largely mirror those observed for the SATT, supporting the idea that TWM-INLA models can also be used to infer heterogeneous effects at the area level.

Finally, since the spatiotemporal interactions considered here rely on first-order neighbors to estimate spatially structured effects, areas with few untreated neighbors in the post-intervention period may yield less accurate estimates. Table A7 presents results on how the proportion of untreated neighbors affects performance. As expected, area-specific effect estimates in treated units improve when a higher share of untreated neighbors is available, provided spatiotemporal dependence exists. In such settings, it may be beneficial to adopt spatial structures that incorporate higher-order (more remote) neighbors (Laffan et al. 2011).

C Latent factor models

Latent factor models (e.g., generalized synthetic controls; Xu 2017) are widely used to accommodate non-parallel trends by decomposing them into time-varying common factors ($F_{t,k}$) with unit-specific loadings ($\Lambda_{i,k}$). These are typically represented as

$$\sum_{k=1}^{K} F_{t,k}\Lambda_{i,k}. \tag{8}$$

A natural question is whether this structure can be incorporated into our spatiotemporal framework to allow for spatially smoothed factor loadings to allow for generalized synthetic controls in settings with residual spatiotemporal dependence (in Γ_{it}). By linear projection, the loadings can be decomposed as follows:

$$\sum_{k=1}^{K} F_{t,k}\Lambda_{i,k} = \sum_{k=1}^{K} F_{t,k}(\beta_k \bar{A}_i + \psi_{i,k}) = \sum_{k=1}^{K} F_{t,k}\beta_k \bar{A}_i + \sum_{k=1}^{K} F_{t,k}\psi_{i,k}, \tag{9}$$

where $\bar{A}_i$ is the unit-level treatment average (Mundlak projection), β_k are the associated coefficients, and $\psi_{i,k}$ are the residualized loadings. The first term acts as a Mundlak correction, which must be included in the model's fixed component ($\mathbf{X}\beta$) to preserve causal identification under the same assumptions as generalized synthetic control models.

A key challenge lies in estimating the factors while ensuring their associated uncertainty propagates through the full model. In the generalized synthetic control method, the number of factors K is selected via cross-validation, and the factors are extracted from the never-treated units Ω_{NT}. To estimate prediction errors, a leave-one-out bootstrap is applied: each control unit is iteratively treated as a pseudo-treated unit to generate an empirical error distribution (Xu 2017). While this approach could, in principle, be adapted to our setting, the computational demands of Bayesian models render such procedures prohibitively slow.

Pang et al. (2022) propose a fully Bayesian dynamic latent factor model (non-spatial), estimated via Markov Chain Monte Carlo methods, with the number of

factors learned via shrinkage priors. In a different context unrelated to synthetic controls, Shin and Ferreira (2023) develop an ICAR-based spatiotemporal factor model that could possibly be adapted to estimate fully Bayesian synthetic controls with spatially structured loadings, although doing so requires additional coding and development beyond the scope of the present work. As a simple proof-of-concept, we here explore a simple two-stage alternative using INLA. Since R-INLA does not support latent factor models natively, we use the following procedure to ensure that uncertainty is propagated through all stages:

1. Resample the N_c never-treated control areas with replacement m times.
2. In each resample, fit a model with unit- and time fixed effects to the $N_c \times T$ outcome matrix $\mathbf{Y}(\mathbf{0})_{\Omega_{\mathrm{NT}}}$ and extract the residual matrix $\tilde{\mathbf{Y}}(\mathbf{0})_{\Omega_{\mathrm{NT}}}$.
3. Extract K latent factors from $\tilde{\mathbf{Y}}(\mathbf{0})_{\Omega_{\mathrm{NT}}}$ using factor analysis.
4. Use INLA to fit m spatiotemporal models to the not-yet and never-treated observations (Ω_0), using the extracted factors as covariates, including area-level random effects for factor loadings and fixed Mundlak corrections.
5. Pool posterior predictions from the m models.

We simulate data following Scenario 2 from Section B.4, but introduce two latent factors representing smooth nonlinear trends. These are generated using a cubic spline basis with four degrees of freedom, where coefficients are drawn independently from a standard Gaussian distribution. The corresponding loadings are generated with spatial dependence using an ICAR model:

$$\Lambda_{i,k} \sim \mathrm{ICAR}(\mathbf{Q}_{\mathrm{ICAR}}), \qquad \text{with } \mathbb{E}[\Lambda_{i,k}] = 0, \ \mathrm{SD} = 1. \tag{10}$$

To induce confounding by the latent factors, we also include the loadings in the treatment assignment model:

$$\mathrm{logit}(\pi_i) = \alpha_i + \sum_{k=1}^{K} \Lambda_{i,k}. \tag{11}$$

We compare the proposed spatiotemporal model with a conventional DID estimator and the generalized synthetic control estimator of Xu (2017). We fit and pool $m = 5$ spatiotemporal models following the procedure described above. We use the factor analysis algorithms implemented in the `gsynth` package for R to perform steps 2-3 on the resampled control data. For computational efficiency, we carry over the estimated number of cross-validated factors from the generalized synthetic control model fit to the main (non-resampled) dataset.

We evaluate two settings: one without spatiotemporal interactions (i.e., no Γ_{it} term) and one with. As shown in Table A1, Panel A, the spatiotemporal factor model produces estimates comparable to those from generalized synthetic controls when no spatiotemporal interactions are present. When such interactions are included, the spatiotemporal model demonstrates superior precision (similar bias, lower RMSE; Table A1, Panel B).

Table A1: Comparison of difference-in-differences, generalized synthetic controls, and INLA models with latent factor terms.

Model	Bias	RMSE	95%CI width	95%CI cov.
A. Without spatiotemporal interactions				
DID	1.81	13.89	10.13	0.34
GSCM	-0.13	0.54	1.62	0.96
INLA-Factor	-0.11	0.57	1.48	0.94
B. With spatiotemporal interactions				
DID	-0.67	12.14	8.94	0.29
GSCM	-0.06	0.81	1.64	0.90
INLA-Factor	-0.07	0.67	1.47	0.92

Notes: DID: Frequentist difference-in-differences model with area and time fixed effects and area-level cluster-robust standard errors; GSCM: Frequentist generalized synthetic control model with number of factors identified using the cross-validation scheme developed by Xu (2017) and parametric bootstrap with 200 resamples for inference; INLA-Factor: $m = 5$ pooled Bayesian models with spatiotemporal random effects estimated using integrated nested Laplace approximation (INLA), including Mundlak terms for unit, time, and latent factor variables in the fixed model component. The true number of latent factors is 2. Panel A shows a scenario without spatiotemporal interactions. Panel B shows a scenario with an AR(1)$\times$ICAR interaction term. The target parameter for bias, root mean squared error (RMSE), and 95% confidence/credible (CI) coverage (95%CI cov.) is the sample average treatment effect on the treated. Values presented are based on 100 replications of each scenario. Results are based on simulations with $N = 50$ areas, $T = 20$ time points, and $N_1 = 10$ treated areas treated simultaneously at $T = 10$.

D Tables

Table A2: Simulation results with homogeneous effects and simultaneous treatment adoption (50 areas, 10 treated).

		Scenario 1: Unstructured data				Scenario 2: Spatiotemporal dependence and parallel trends				Scenario 3: Spatially varying, non-parallel trends			
Model	T	Bias	RMSE	95%CI width	95%CI cov.	Bias	RMSE	95%CI width	95%CI cov.	Bias	RMSE	95%CI width	95%CI cov.
OLS	6	0.38	0.74	1.13	0.51	0.37	0.80	1.15	0.51	1.38	1.72	1.90	0.31
DID	6	-0.02	0.30	1.14	0.93	0.00	0.36	0.99	0.82	0.66	0.91	1.41	0.54
DID, Linear trend	6	-0.04	0.57	2.55	0.98	-0.01	0.49	2.02	0.96	-0.01	0.52	2.02	0.96
INLA, non-TWM	6	0.14	0.34	1.10	0.90	0.08	0.28	0.98	0.91	0.45	0.68	1.97	0.88
INLA, TWM	6	-0.02	0.30	1.24	0.95	0.01	0.30	1.07	0.94	0.07	0.59	2.35	0.95
INLA, TWFE	6	-0.01	0.30	1.30	0.91	-0.01	0.32	0.94	0.85	0.06	0.61	2.22	0.91
OLS	10	0.37	0.66	0.93	0.51	0.39	0.75	0.92	0.46	1.76	2.23	2.21	0.29
DID	10	0.01	0.23	0.90	0.95	0.03	0.29	0.82	0.83	0.98	1.34	1.48	0.35
DID, Linear trend	10	0.04	0.48	1.88	0.95	0.01	0.42	1.63	0.94	0.04	0.46	1.62	0.92
INLA, non-TWM	10	0.12	0.28	0.87	0.91	0.15	0.29	0.93	0.91	0.35	0.64	2.09	0.88
INLA, TWM	10	0.01	0.24	0.96	0.95	0.04	0.24	1.02	0.95	0.07	0.53	2.26	0.95
INLA, TWFE	10	0.01	0.23	1.02	0.91	0.04	0.25	0.76	0.84	0.05	0.52	2.12	0.94
OLS	20	0.41	0.67	0.69	0.40	0.39	0.69	0.69	0.41	2.93	3.64	2.85	0.22
DID	20	0.00	0.17	0.64	0.92	0.00	0.26	0.61	0.80	1.86	2.45	1.82	0.22
DID, Linear trend	20	0.05	0.32	1.31	0.96	-0.01	0.44	1.22	0.86	0.00	0.40	1.21	0.87
INLA, non-TWM	20	0.08	0.20	0.65	0.90	0.11	0.25	0.78	0.89	0.21	0.53	2.13	0.96
INLA, TWM	20	0.00	0.17	0.69	0.94	0.00	0.21	0.87	0.94	0.05	0.47	2.18	0.98
INLA, TWFE	20	0.00	0.17	0.71	0.93	-0.01	0.21	0.71	0.88	0.04	0.45	1.93	0.97

Notes: OLS: Ordinary least squares model with a treatment dummy as the only left-hand side variable; DID: Frequentist difference-in-differences model with area and time fixed effects; DID, Linear trend: Frequentist DID model with area-specific linear trends; INLA, TWM: Bayesian model with spatiotemporal random effects estimated using integrated nested Laplace approximation (INLA), including a two-way Mundlak (TWM) specification in the fixed model component; INLA, non-TWM: Bayesian spatiotemporal model without a two-way Mundlak specification (i.e., relying only on random effects); INLA, TWFE: Bayesian spatiotemporal model with area and time fixed effects. Specification of the random effects in the spatiotemporal models vary by scenario (the random component is correctly specified in all fitted INLA models; see text for details). The target parameter for bias, root mean squared error (RMSE), and 95% confidence/credible (CI) coverage (95%CI cov.) is the sample average treatment effect on the treated. Values presented are based on 200 replications of each scenario.

Table A3: Simulation results with heterogeneous effects and simultaneous treatment adoption (50 areas, 10 treated).

		Scenario 1: Unstructured data				Scenario 2: Spatiotemporal dependence and parallel trends				Scenario 3: Spatially varying, non-parallel trends			
Model	T	Bias	RMSE	95%CI width	95%CI cov.	Bias	RMSE	95%CI width	95%CI cov.	Bias	RMSE	95%CI width	95%CI cov.
OLS	6	0.43	0.77	1.57	0.69	0.41	0.80	1.57	0.66	1.28	1.62	2.20	0.44
DID	6	-0.02	0.28	1.28	0.99	0.02	0.34	1.15	0.91	0.60	0.85	1.46	0.56
DID, Linear trend	6	0.72	0.97	2.72	0.83	0.72	0.87	2.28	0.77	0.69	0.85	2.24	0.85
INLA, non-TWM	6	0.14	0.34	1.07	0.88	0.11	0.29	0.99	0.89	0.42	0.66	1.98	0.85
INLA, TWM	6	-0.02	0.29	1.23	0.97	0.02	0.29	1.09	0.94	0.03	0.51	2.33	0.98
INLA, TWFE	6	-0.02	0.28	1.28	0.94	0.02	0.30	0.99	0.87	0.01	0.56	2.20	0.93
OLS	10	0.39	0.75	1.31	0.61	0.36	0.70	1.33	0.67	1.97	2.42	2.30	0.24
DID	10	0.03	0.23	1.04	0.97	0.01	0.33	0.99	0.86	1.11	1.42	1.51	0.30
DID, Linear trend	10	1.31	1.39	2.06	0.28	1.30	1.37	1.89	0.22	1.34	1.44	1.90	0.22
INLA, non-TWM	10	0.14	0.28	0.88	0.88	0.11	0.24	0.93	0.94	0.35	0.66	2.07	0.87
INLA, TWM	10	0.04	0.24	0.98	0.95	0.02	0.25	1.02	0.96	0.05	0.56	2.27	0.96
INLA, TWFE	10	0.03	0.24	0.99	0.92	0.01	0.28	0.78	0.83	0.03	0.56	2.06	0.94
OLS	20	0.48	0.69	1.10	0.55	0.45	0.74	1.09	0.52	3.13	4.00	2.81	0.22
DID	20	0.01	0.14	0.86	1.00	0.02	0.27	0.84	0.91	2.02	2.67	1.80	0.23
DID, Linear trend	20	2.53	2.57	1.62	0.00	2.50	2.55	1.56	0.00	2.48	2.54	1.57	0.00
INLA, non-TWM	20	0.09	0.18	0.66	0.95	0.15	0.28	0.80	0.84	0.19	0.58	2.18	0.94
INLA, TWM	20	0.01	0.15	0.70	0.98	0.03	0.22	0.87	0.94	0.02	0.52	2.25	0.98
INLA, TWFE	20	0.01	0.15	0.67	0.98	0.02	0.21	0.69	0.88	0.01	0.54	1.96	0.93

Notes: OLS: Ordinary least squares model with a treatment dummy as the only left-hand side variable; DID: Frequentist difference-in-differences model with area and time fixed effects; DID, Linear trend: Frequentist DID model with area-specific linear trends; INLA, TWM: Bayesian model with spatiotemporal random effects estimated using integrated nested Laplace approximation (INLA), including a two-way Mundlak (TWM) specification in the fixed model component; INLA, non-TWM: Bayesian spatiotemporal model without a two-way Mundlak specification (i.e., relying only on random effects); INLA, TWFE: Bayesian spatiotemporal model with area and time fixed effects. Specification of the random effects in the spatiotemporal models vary by scenario (the random component is correctly specified in all fitted INLA models; see text for details). The target parameter for bias, root mean squared error (RMSE), and 95% confidence/credible (CI) coverage (95%CI cov.) is the sample average treatment effect on the treated. Values presented are based on 200 replications of each scenario.

Table A4: Simulation results with heterogeneous effects and staggered treatment adoption (50 areas, 10 treated).

		Scenario 1: Unstructured data				Scenario 2: Spatiotemporal dependence and parallel trends				Scenario 3: Spatially varying, non-parallel trends			
Model	T	Bias	RMSE	95%CI width	95%CI cov.	Bias	RMSE	95%CI width	95%CI cov.	Bias	RMSE	95%CI width	95%CI cov.
OLS	6	0.43	0.86	1.83	0.68	0.35	0.95	1.84	0.65	1.24	1.70	2.62	0.50
DID	6	0.08	0.32	1.40	0.98	0.06	0.33	1.27	0.95	0.60	0.88	1.67	0.62
DID, Linear trend	6	0.37	0.63	2.21	0.92	0.37	0.57	1.79	0.85	0.43	0.63	1.83	0.84
INLA, non-TWM	6	0.16	0.35	1.23	0.92	0.08	0.25	1.04	0.98	0.25	0.51	1.72	0.91
INLA, TWM	6	0.01	0.32	1.35	0.96	-0.01	0.26	1.12	0.97	0.06	0.46	1.89	0.97
INLA, TWFE	6	0.02	0.32	1.35	0.96	0.01	0.26	1.00	0.92	0.06	0.45	1.73	0.92
OLS	10	0.42	0.78	1.61	0.67	0.47	0.87	1.60	0.67	1.59	2.20	3.07	0.46
DID	10	0.09	0.28	1.18	0.95	0.12	0.36	1.11	0.88	0.89	1.23	1.85	0.54
DID, Linear trend	10	0.58	0.71	1.70	0.76	0.58	0.74	1.52	0.66	0.60	0.77	1.55	0.63
INLA, non-TWM	10	0.11	0.31	1.02	0.89	0.09	0.29	0.99	0.91	0.11	0.48	1.81	0.92
INLA, TWM	10	0.00	0.29	1.08	0.93	0.00	0.27	1.07	0.93	-0.04	0.48	1.91	0.96
INLA, TWFE	10	0.00	0.28	1.06	0.92	0.02	0.28	0.90	0.85	-0.03	0.46	1.75	0.94
OLS	20	0.39	0.75	1.38	0.60	0.38	0.76	1.36	0.65	3.41	4.46	4.13	0.31
DID	20	0.16	0.26	1.00	0.99	0.17	0.34	0.99	0.85	2.09	2.71	2.38	0.29
DID, Linear trend	20	1.04	1.13	1.35	0.19	1.11	1.22	1.27	0.17	1.00	1.11	1.29	0.27
INLA, non-TWM	20	0.06	0.21	0.77	0.94	0.10	0.26	0.87	0.88	0.08	0.45	1.89	0.95
INLA, TWM	20	-0.01	0.19	0.80	0.98	0.01	0.23	0.92	0.95	-0.03	0.42	1.92	0.96
INLA, TWFE	20	-0.01	0.19	0.79	0.97	0.00	0.23	0.75	0.86	-0.01	0.42	1.70	0.95

Notes: OLS: Ordinary least squares model with a treatment dummy as the only left-hand side variable; DID: Frequentist difference-in-differences model with area and time fixed effects; DID, Linear trend: Frequentist DID model with area-specific linear trends; INLA, TWM: Bayesian model with spatiotemporal random effects estimated using integrated nested Laplace approximation (INLA), including a two-way Mundlak (TWM) specification in the fixed model component; INLA, non-TWM: Bayesian spatiotemporal model without a two-way Mundlak specification (i.e., relying only on random effects); INLA, TWFE: Bayesian spatiotemporal model with area and time fixed effects. Specification of the random effects in the spatiotemporal models vary by scenario (the random component is correctly specified in all fitted INLA models; see text for details). The target parameter for bias, root mean squared error (RMSE), and 95% confidence/credible (CI) coverage (95%CI cov.) is the sample average treatment effect on the treated. Values presented are based on 200 replications of each scenario.

Table A5: Simulation results with a single treated unit (50 areas, 1 treated).

Model	T	Scenario 1: Unstructured data				Scenario 2: Spatiotemporal dependence and parallel trends				Scenario 3: Spatially varying, non-parallel trends			
		Bias	RMSE	95%CI width	95%CI cov.	Bias	RMSE	95%CI width	95%CI cov.	Bias	RMSE	95%CI width	95%CI cov.
OLS	6	0.47	1.28	2.22	0.59	0.35	1.36	2.08	0.55	1.18	2.90	2.26	0.29
DID	6	0.07	0.77	2.68	0.85	0.08	1.07	2.26	0.66	0.71	1.87	2.56	0.49
DID, Linear trend	6	0.11	1.63	4.91	0.78	0.01	1.41	4.40	0.79	0.14	1.37	4.14	0.74
INLA, non-TWM	6	0.18	0.77	3.09	0.95	0.07	0.80	2.81	0.93	0.39	1.47	5.65	0.95
INLA, TWM	6	0.07	0.79	3.52	0.97	0.01	0.85	3.09	0.91	0.15	1.54	6.68	0.96
INLA, TWFE	6	0.06	0.78	3.49	0.94	0.08	0.89	2.71	0.84	0.06	1.63	6.31	0.93
OLS	10	0.30	1.21	2.13	0.61	0.42	1.22	1.87	0.51	1.96	4.23	2.25	0.18
DID	10	-0.05	0.72	2.41	0.89	0.11	0.88	1.99	0.74	1.09	2.76	2.65	0.31
DID, Linear trend	10	-0.15	1.34	4.46	0.86	0.26	1.33	3.72	0.83	-0.05	1.41	3.94	0.82
INLA, non-TWM	10	0.03	0.67	2.48	0.93	0.17	0.64	2.66	0.96	0.23	1.65	5.78	0.94
INLA, TWM	10	-0.05	0.74	2.74	0.94	0.10	0.73	3.03	0.96	-0.03	1.64	6.30	0.94
INLA, TWFE	10	-0.04	0.73	2.88	0.93	0.09	0.73	2.40	0.88	-0.04	1.67	5.90	0.92
OLS	20	0.52	1.08	1.70	0.54	0.36	1.28	1.59	0.51	2.65	7.11	2.28	0.12
DID	20	0.00	0.44	1.74	0.93	0.04	0.69	1.57	0.75	1.48	4.53	2.72	0.14
DID, Linear trend	20	0.08	0.86	3.38	0.93	0.03	1.17	3.06	0.79	-0.09	1.06	3.01	0.80
INLA, non-TWM	20	0.07	0.48	1.84	0.95	0.06	0.57	2.18	0.94	-0.02	1.24	5.72	0.98
INLA, TWM	20	0.00	0.46	1.94	0.97	-0.01	0.57	2.39	0.97	-0.12	1.24	5.93	0.99
INLA, TWFE	20	0.00	0.47	2.07	0.94	0.01	0.62	1.74	0.84	-0.13	1.26	5.40	0.96

Notes: OLS: Ordinary least squares model with a treatment dummy as the only left-hand side variable; DID: Frequentist difference-in-differences model with area and time fixed effects; DID, Linear trend: Frequentist DID model with area-specific linear trends; INLA, TWM: Bayesian model with spatiotemporal random effects estimated using integrated nested Laplace approximation (INLA), including a two-way Mundlak (TWM) specification in the fixed model component; INLA, non-TWM: Bayesian spatiotemporal model without a two-way Mundlak specification (i.e., relying only on random effects); INLA, TWFE: Bayesian spatiotemporal model with area and time fixed effects. Specification of the random effects in the spatiotemporal models vary by scenario (the random component is correctly specified in all fitted INLA models; see text for details). The target parameter for bias, root mean squared error (RMSE), and 95% confidence/credible (CI) coverage (95%CI cov.) is the sample average treatment effect on the treated. Values presented are based on 200 replications of each scenario.

Table A6: Simulation results for area-specific post-intervention average effects (50 areas, 10 treated).

		Scenario 1: Unstructured data				Scenario 2: Spatiotemporal dependence and parallel trends				Scenario 3: Spatially varying, non-parallel trends			
Model	T	Bias	RMSE	95%CI width	95%CI cov.	Bias	RMSE	95%CI width	95%CI cov.	Bias	RMSE	95%CI width	95%CI cov.
A. Homogeneous effects													
INLA, non-TWM	6	0.14	0.82	3.14	0.94	0.08	0.77	2.78	0.94	0.45	1.40	4.39	0.89
INLA, TWM	6	-0.02	0.80	3.17	0.94	0.01	0.77	2.78	0.93	0.07	1.37	4.57	0.91
INLA, TWFE	6	-0.01	0.87	3.72	0.92	-0.01	0.83	2.68	0.86	0.06	1.34	4.42	0.89
INLA, non-TWM	10	0.12	0.67	2.49	0.94	0.15	0.69	2.66	0.95	0.35	1.56	5.13	0.91
INLA, TWM	10	0.01	0.64	2.54	0.95	0.04	0.66	2.65	0.95	0.07	1.52	5.15	0.91
INLA, TWFE	10	0.01	0.67	2.92	0.93	0.04	0.75	2.18	0.83	0.05	1.37	5.15	0.94
INLA, non-TWM	20	0.08	0.51	1.85	0.93	0.11	0.61	2.20	0.92	0.21	1.56	5.77	0.94
INLA, TWM	20	0.00	0.48	1.88	0.94	0.00	0.59	2.22	0.92	0.05	1.47	5.75	0.95
INLA, TWFE	20	0.00	0.48	2.02	0.94	-0.01	0.60	2.00	0.88	0.04	1.37	5.21	0.94
B. Heterogeneous effects													
INLA, non-TWM	6	0.15	0.81	3.06	0.95	0.11	0.77	2.79	0.93	0.42	1.38	4.39	0.89
INLA, TWM	6	-0.01	0.81	3.13	0.95	0.02	0.79	2.84	0.93	0.03	1.33	4.54	0.91
INLA, TWFE	6	-0.02	0.85	3.66	0.94	0.02	0.84	2.82	0.87	0.01	1.29	4.47	0.90
INLA, non-TWM	10	0.14	0.65	2.50	0.94	0.10	0.70	2.64	0.94	0.35	1.58	5.13	0.90
INLA, TWM	10	0.04	0.64	2.56	0.96	0.02	0.71	2.65	0.93	0.05	1.56	5.17	0.91
INLA, TWFE	10	0.03	0.66	2.83	0.93	0.01	0.79	2.22	0.81	0.03	1.42	5.06	0.93
INLA, non-TWM	20	0.09	0.49	1.87	0.95	0.15	0.62	2.25	0.92	0.19	1.53	5.87	0.95
INLA, TWM	20	0.01	0.48	1.88	0.95	0.03	0.61	2.25	0.93	0.02	1.48	5.88	0.95
INLA, TWFE	20	0.01	0.47	1.91	0.94	0.02	0.59	1.96	0.88	0.01	1.40	5.27	0.94

Notes: INLA, TWM: Bayesian model with spatiotemporal random effects estimated using integrated nested Laplace approximation (INLA), including a two-way Mundlak (TWM) specification in the fixed model component; INLA, non-TWM: Bayesian spatiotemporal model without a two-way Mundlak specification (i.e., relying only on random effects); INLA, TWFE: Bayesian spatiotemporal model with area and time fixed effects. Specification of the random effects in the spatiotemporal models vary by scenario (the random component is correctly specified in all fitted INLA models; see text for details). The target parameter for bias, root mean squared error (RMSE), and 95% credible (CI) coverage (95%CI cov.) is the area-specific post-intervention treatment effect. Values presented are metrics averaged over the ten treated units and based on 200 replications of each scenario. All estimates are from simulations with simultaneous treatment adoption.

Table A7: Simulation results for area-specific post-intervention average effects by groups of treated areas defined by the share of their direct spatial neighbors that remain untreated throughout the entire study period (50 areas, 10 treated).

		Scenario 1: Unstructured data				Scenario 2: Spatiotemporal dependence and parallel trends				Scenario 3: Spatially varying, non-parallel trends			
Model	T	Bias	RMSE	95%CI width	95%CI cov.	Bias	RMSE	95%CI width	95%CI cov.	Bias	RMSE	95%CI width	95%CI cov.
A. Treated areas with $\leq$ 50% *untreated neighbors*													
INLA, non-TWM	6	0.26	0.89	3.13	0.93	0.26	0.95	2.96	0.89	0.88	1.67	4.78	0.83
INLA, TWM	6	0.17	0.93	3.21	0.93	0.14	0.97	3.02	0.89	0.47	1.42	4.87	0.91
INLA, TWFE	6	0.03	0.94	3.71	0.93	0.04	1.00	2.79	0.81	0.50	1.43	4.68	0.86
INLA, non-TWM	10	0.42	0.79	2.60	0.93	0.22	0.70	2.74	0.94	0.14	1.74	5.48	0.88
INLA, TWM	10	0.29	0.68	2.56	0.96	0.18	0.72	2.78	0.92	-0.12	1.77	5.54	0.88
INLA, TWFE	10	0.22	0.74	2.92	0.91	0.20	0.85	2.43	0.80	-0.21	1.66	5.36	0.90
INLA, non-TWM	20	0.15	0.47	1.93	0.95	0.21	0.65	2.41	0.92	0.16	1.81	6.44	0.94
INLA, TWM	20	0.04	0.44	1.93	0.97	0.07	0.67	2.32	0.92	-0.03	1.64	6.50	0.96
INLA, TWFE	20	0.00	0.45	1.93	0.96	-0.02	0.66	2.09	0.90	-0.18	1.53	5.75	0.95
B. Treated areas with $>$ 50% *untreated neighbors*													
INLA, non-TWM	6	0.14	0.81	3.06	0.95	0.10	0.76	2.78	0.93	0.39	1.37	4.37	0.90
INLA, TWM	6	-0.02	0.80	3.13	0.95	0.01	0.78	2.83	0.93	0.01	1.32	4.52	0.91
INLA, TWFE	6	-0.02	0.84	3.66	0.94	0.02	0.83	2.82	0.87	-0.01	1.28	4.46	0.90
INLA, non-TWM	10	0.13	0.64	2.50	0.94	0.10	0.70	2.64	0.94	0.36	1.57	5.11	0.90
INLA, TWM	10	0.02	0.63	2.56	0.96	0.01	0.71	2.64	0.93	0.06	1.54	5.16	0.91
INLA, TWFE	10	0.02	0.66	2.82	0.93	0.00	0.78	2.21	0.81	0.05	1.41	5.05	0.93
INLA, non-TWM	20	0.08	0.49	1.87	0.95	0.14	0.61	2.24	0.92	0.19	1.51	5.84	0.95
INLA, TWM	20	0.01	0.48	1.88	0.95	0.02	0.60	2.25	0.93	0.02	1.47	5.84	0.95
INLA, TWFE	20	0.02	0.47	1.91	0.94	0.02	0.58	1.95	0.88	0.02	1.39	5.24	0.94

Notes: INLA, TWM: Bayesian model with spatiotemporal random effects estimated using integrated nested Laplace approximation (INLA), including a two-way Mundlak (TWM) specification in the fixed model component; INLA, non-TWM: Bayesian spatiotemporal model without a two-way Mundlak specification (i.e., relying only on random effects); INLA, TWFE: Bayesian spatiotemporal model with area and time fixed effects. Specification of the random effects in the spatiotemporal models vary by scenario (the random component is correctly specified in all fitted INLA models; see text for details). The target parameter for bias, root mean squared error (RMSE), and 95% credible (CI) coverage (95%CI cov.) is the area-specific post-intervention treatment effect. Values presented are metrics averaged over the treated units within each group and based on 200 replications of each scenario. All estimates are from simulations with simultaneous adoption and heterogeneous treatment effects.

References

Besag, J.: 1974, Spatial interaction and the statistical analysis of lattice systems, *Journal of the Royal Statistical Society: Series B (Methodological)* **36**(2), 192–225.

Borusyak, K., Jaravel, X. and Spiess, J.: 2024, Revisiting event-study designs: robust and efficient estimation, *Review of Economic Studies* **91**(6), 3253–3285.

Cameron, A. C. and Miller, D. L.: 2015, A practitioner's guide to cluster-robust inference, *Journal of human resources* **50**(2), 317–372.

Conley, T. G. and Taber, C. R.: 2011, Inference with "difference in differences" with a small number of policy changes, *The Review of Economics and Statistics* **93**(1), 113–125.

Goicoa, T., Adin, A., Ugarte, M. and Hodges, J.: 2018, In spatio-temporal disease mapping models, identifiability constraints affect pql and inla results, *Stochastic Environmental Research and Risk Assessment* **32**, 749–770.

Goodman-Bacon, A.: 2021, Difference-in-differences with variation in treatment timing, *Journal of econometrics* **225**(2), 254–277.

Knorr-Held, L.: 2000, Bayesian modelling of inseparable space-time variation in disease risk, *Statistics in medicine* **19**(17-18), 2555–2567.

Laffan, S. W., Wang, Z. and Ward, M. P.: 2011, The effect of neighbourhood definitions on spatio-temporal models of disease outbreaks: separation distance versus range overlap, *Preventive veterinary medicine* **102**(3), 218–229.

Lindgren, F. and Rue, H.: 2015, Bayesian spatial modelling with r-inla, *Journal of statistical software* **63**(19).

Pang, X., Liu, L. and Xu, Y.: 2022, A bayesian alternative to synthetic control for comparative case studies, *Political Analysis* **30**(2), 269–288.

Rue, H. and Held, L.: 2005, *Gaussian Markov random fields: theory and applications*, Chapman and Hall/CRC.

Shin, H. and Ferreira, M. A.: 2023, Dynamic icar spatiotemporal factor models, *Spatial Statistics* **56**, 100763.

Xu, Y.: 2017, Generalized synthetic control method: Causal inference with interactive fixed effects models, *Political Analysis* **25**(1), 57–76.
